# Lateral rectus muscle differentiation potential in paralytic esotropia patients

**DOI:** 10.1186/s12886-021-01994-4

**Published:** 2021-05-27

**Authors:** Qing Xia, Xiangtian Ling, Zhonghao Wang, Tao Shen, Minghao Chen, Danyi Mao, Xinqi Ma, Jie Ning, Han Zhang, Dongli Chen, Qiong Gu, Huangxuan Shen, Jianhua Yan

**Affiliations:** 1 State Key Laboratory of Ophthalmology, Zhongshan Ophthalmic Center, Sun Yat-sen University, 54 Xianlie Road, Guangzhou, 510060 China; 2 Research Center for Drug Discovery, School of Pharmaceutical Sciences, Sun Yat-sen University, Guangzhou, 510006 People’s Republic of China; 3 Biobank of Eye, State Key Laboratory of Ophthalmology, Zhongshan Ophthalmic Center, Sun Yat-sen University, 54 Xianlie Road, Guangzhou, 510060 China

**Keywords:** Strabismus surgery, Paralytic esotropia, Muscle satellite cells, Extraocular muscle, Muscle differentiation

## Abstract

**Purpose and background:**

Recently, we found that maximal medial rectus recession and lateral rectus resection in patients with complete lateral rectus paralysis resulted in a partial restoration of abduction. In an attempt to understand some of the mechanisms involved with this effect we examined gene expression profiles of lateral recti from these patients, with our focus being directed to genes related to myogenesis.

**Materials and methods:**

Lateral recti resected from patients with complete lateral rectus paralysis and those from concomitant esotropia (controls) were collected. Differences in gene expression profiles between these two groups were examined using microarray analysis and quantitative Reverse-transcription PCR (qRT-PCR).

**Results:**

A total of 3056 differentially expressed genes (DEGs) were identified between these two groups. Within the paralytic esotropia group, 2081 genes were up-regulated and 975 down-regulated. The results of RT-PCR revealed that PAX7, MYOG, PITX1, SIX1 and SIX4 showed higher levels of expression, while that of MYOD a lower level of expression within the paralytic esotropia group as compared with that in the control group (*p* < 0.05).

**Conclusion:**

The decreased expression of MYOD in the paralytic esotropia group suggested that extraocular muscle satellite cell (EOMSCs) differentiation processes were inhibited. Whereas the high expression levels of PAX7, SIX1/4 and MYOG, suggested that the EOMSCs were showing an effective potential for differentiation. The stimulation resulting from muscle surgery may induce EOMSCs to differentiate and thus restore abduction function.

**Supplementary Information:**

The online version contains supplementary material available at 10.1186/s12886-021-01994-4.

## Introduction

Complete lateral rectus paralysis is often due to disorders of the sixth cranial nerve, resulting in paralytic esotropia, absence of abduction and a compensatory head position directed toward the affected side. In the early stages of this disorder, local injection of botulinum toxin A, wearing corrective prisms or other conservative treatments can be applied. At later stages, treatments often require surgical correction involving transposition of the superior and inferior rectus muscles within the affected eyes, which may be combined with medial rectus recession [1]. However, restoration of abduction after such surgery is problematic [2, 3]. Recently, we have found that a complete medial rectus recession combined with lateral rectus resection not only achieved orthotopic eye position, but also restored abduction function in some patients. While the underlying mechanisms for this effect remain unknown, it has been reported that increased satellite cell proliferation as well as rapid integration of these cells into myofibers is observed after either stretching or shortening of rectus muscles in rabbits [4]. Such findings raise the issue as to whether the stimulation resulting from this surgery induces proliferation of extraocular muscle satellite cells to restore abduction within these affected eyes.

To address this issue, an assessment of the molecular background of the affected lateral rectus, especially as related to the proliferation and differentiation of extraocular muscle satellite cells is essential. Currently, no such studies exist on extraocular muscle satellite cell gene expression in these subjects. Therefore, we investigated the gene expression profile with use of microarray analysis and reverse-transcription PCR (RT-PCR) in lateral recti resected from patients with complete sixth cranial nerve paralysis. The lateral rectus of concomitant esotropia patients with normal extraocular muscles [5, 6] served as controls. Thus, the purpose of this study was to examine potential differences in gene expression within lateral rectus muscles between these two groups. Such information will significantly aid in the understanding of possible molecular mechanisms involved with functional recovery of lateral rectus muscles after horizontal rectus recession and resection in patients with complete lateral rectus paralysis.

## Materials and methods

### Samples

Resected lateral recti were obtained from 16 patients with complete lateral rectus palsy and 26 cases of concomitant esotropia requiring rectus resection for strabismus correction. Samples consisted of lateral recti muscles containing both muscle and tendon. This study was conducted over the period from January 2016 to December 2018 at the Zhongshan Ophthalmic Center, Guangzhou, China. Informed consent adhering to the tenets of the Declaration of Helsinki was obtained from all participants or their guardians prior to the study and this study was approved by the Institutional Review Board of the Zhongshan Ophthalmic Center.

### Microarray data

The Affymetrix Human Genome U133 Plus 2.0 Array (Affymetrix; Thermo Fisher Scientific, Inc., Waltham, MA, USA) was used to perform microarray analyses of the 3 pairs of muscle samples which were precisely matched for age, gender and muscle length resected. Significance analysis of microarray (SAM) was used to identify genes that were differentially expressed. SAM identifies genes with statistically significant changes in expression by assimilating a set of gene-specific t-tests and provides an estimate of the false discovery rate (FDR) from generated data by performing premutation calculations.

### Screening of DEGs

If one gene was detected by multiple probes, the mean value of the probes can then represent the expression level [7]. Gene expression data from the paralytic and concomitant esotropia groups were separated according to the Linear Models for Microarray Data package (LIMMA), and DEGs between the two groups were screened in R software [8]. The criterion for detection of DEGs required that a *P*-value < 0.05 and |log2FC| ≥2 were obtained in the screening. The heat-map was then plotted as acquired from the heat map data in the R package of DEGs.

### Bioinformatics analyses and functional enrichment

Gene Ontology (GO) functional enrichment and Kyoto Encyclopedia of Genes and Genomes (KEGG) pathway analyses were both obtained in order to analyze gene function and pathways related to the DEGs. Terms were chosen as a standard with a *P*-value of < 0.05 and Fold Change ≥2.

### qRT-PCR (quantitative real time- polymerase chain reaction)

Total RNA extractions were performed on lateral rectus palsy and concomitant esotropia muscle samples according to the TRIZOL instructions. Specific primers were synthesized for 10 selected genes (SIX1, SIX4, PITX1, PAX7, MYF5, MYOD, MYOG, ITGA7, CXCR4, VCAM) related to myogenesis [9, 10]. Real-time PCR was then used to determine relative expression levels of these 10 genes with GAPDH as a control. The primers were synthesized by Synbio technologies (Suzhou, China). The sequence of primers is shown in Table 1.

**Table 1:** Primer sequences

Genes	Primer forward sequences (5′--3′)	Primer reverse sequences(5′--3′)
MYOG	AGCGAATGCAGCTCTCACAG	CATCTGTAGGGTCAGCCGTG
MYF5	GCAGGATGGACGTGATGGAT	CTCGTCCCCAAATTCACCCT
CXCR4	TGGTCTATGTTGGCGTCTGG	GTCATTGGGGTAGAAGCGGT
PAX7	GGACAAGAAGGAGGACGACG	CAGGTTCCGACTCCACATCC
PITX1	AACCGCTACCCCGACATGAG	CACGTAGCCACCCTTGCAC
ITGA7	CAGTGGTTGGGAGTCAGTGT	CTGCCTTGCCTCATATCGGT
MYOD1	GCCACAACGGACGACTTCTA	AGTGCTCTTCGGGTTTCAGG
SIX4	GGCATTGTCCAGATCCCCAA	CTGCACTGGGAGCAAGAGAG
VCAM1	GTGACGAATGAGGGGACCAC	ACACTTGACTGTGATCGGCTT
SIX1	CAACTGGTTTAAGAACCGGAGG	CCGAGTTCTGGTCTGGACTTT

### Statistical analysis

Each experiment was repeated at least three times and the IBM SPSS Statistics 20 software program was used for statistical analysis. Results are displayed as means ± standard deviations, with significance of differences between groups being determined with use of an independent groups t-test and the multiple LSD’s multiple-comparison test (one-way ANOVA). Statistically significant *P* values required that *P*< 0.05 (∗), *P*< 0.01 (∗∗) or *P* < 0.001(∗∗∗) while *P*> 0.05 (#) indicates non-significant differences.

## Results

### A typical case of functional abduction recovery in a patient with complete lateral rectus paralysis

**Fig. 1:**
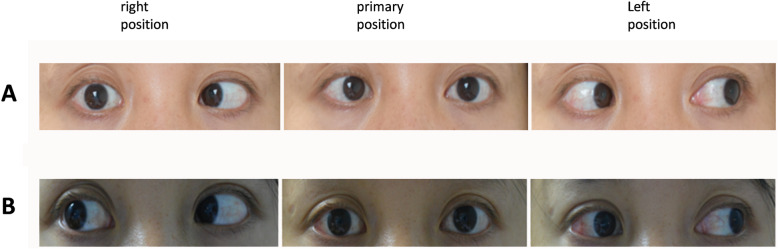
Typical case of a near complete recovery of abduction in a patient with complete sixth cranial nerve paralysis after a serious traumatic fall. **A**. Clinical picture demonstrating that a 35 PD esotropia in the right eye which could not move across the midline before surgery. **B**. Two months after a 7 mm medial rectus recession and a 13 mm lateral rectus resection, the patient showed normal ocular alignment and only − 1 underaction of abduction in the right eye

A 28-year-old female that experienced a coma after a serious fall 20 years prior found that upon recovery from the coma her right eye was displaced inward, along with horizontal diplopia. No improvement was observed following conservative treatment. Visual acuity, anterior segment and fundus were normal in both eyes. She showed a 35 prism diopters (PD) esotropia at distance and 28 PD esotropia at near. No abduction of the right eye was present and her head was positioned at a 46 degree face turn to the right side. Forced duction test revealed no resistance of the right medial rectus. She was diagnosed as complete right sixth cranial nerve (lateral rectus muscle) palsy. She received a 7 mm recession of the medial rectus and 13 mm resection of the lateral rectus in the right eye. One week post-surgery she showed 20 PD exotropia and a − 2 underaction in both abduction and adduction in the right eye. At 6 months post-surgery a normal eye position was present and only − 1 underaction in both abduction and adduction. Her compensatory head position dissipated. She showed a normal 40 s arc of stereopsis at near and 200 s arc of stereopsis at distance by Randot (Fig. 1A, B).

### Expression of differentially-related genes of satellite cells in extraocular muscles

**Fig. 2:**
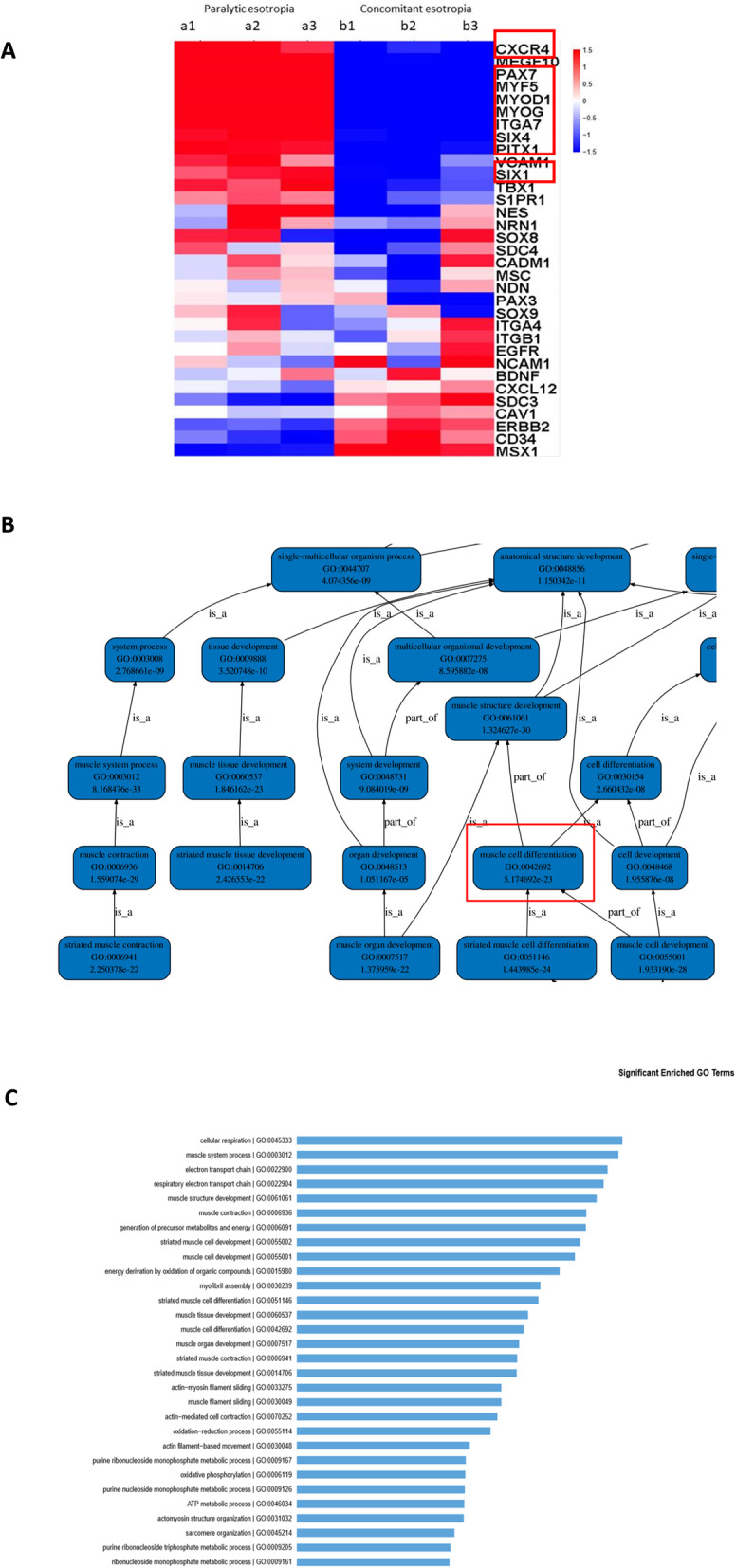
Differential expression of genes in satellite cells of extraocular muscles in the complete lateral rectus paralysis group. **A**. Volcano plot of the differentially expressed genes. The x axis is log2 (fold change) while the y axis is -log10 (*P*-value). Black dots indicate non-differentially expressed genes while the red dots indicate increases in expressed genes and green dots indicate decreases in expressed genes. **B**. Significance Analysis of Microarrays (SAM) of Affymetrix GeneChip® Human Genome U133 Plus 2.0 Array (HG-U133 Plus 2.0) from age and sex matched extraocular muscles of group A (Paralytic esotropia) and group B (Concomitant esotropia). **C**. The upper 30 significant enriched GO terms. The abscissa is −log10 (*p* − Value) and the ordinate is the significant enriched GO terms. Red indicates molecular function terms, blue indicates biological process terms and green indicates cellular component terms

With use of a gene expression microarray 28,049 genes were detected, with 3056 differentially expressed genes (DEGs) between the two groups. Within the paralytic esotropia group, 2081 genes were up-regulated and 975 down-regulated (Fold Change > 2.0) (Fig. 2A). The upper 33 most significant terms were listed, with multiple genes from these terms being related to differentiation of muscle stem cells (Fig. 2B). Groups (*N* = 874) of GO function (*P* < 0.05) and KEGG pathway (*P* < 0.05) enrichment analyses were used to analyze functions associated with DEGs (e.g. GO0003012, 0061061, 0006936, 0055002, 0055001, 0030239, 0060537, 0007517). Multiple sets of genes were associated with the differentiation of extraocular muscle satellite cells (GO0042692). Among these, 30 were related to the function of extraocular muscle satellite cells (Fig. 2C).

### High expressions of SIX1/SIX4, PAX7, MYOG, and a low expression of MYOD was observed in patients with complete lateral rectus paralysis

**Fig. 3:**
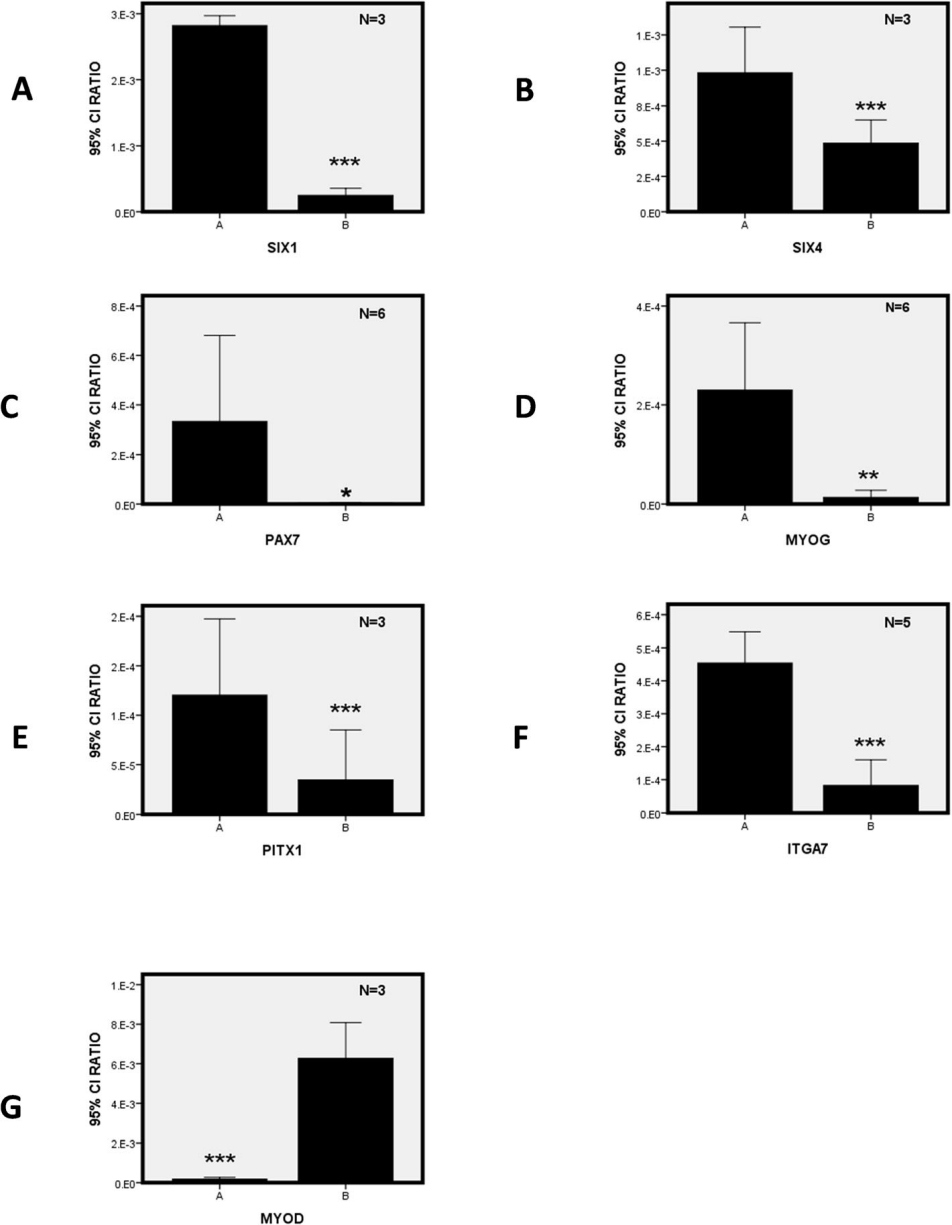
High expressions of SIX1/SIX4, PAX7, MYOG, and low expression of MYOD were observed in patients with complete lateral rectus paralysis*.* Results of the error column diagram were matched: **A** - expression level of SIX1, **B** - expression level of SIX4, **C** - expression level of PAX7, **D** - expression level of MYOG, **E** - expression level of PITX1 and **F** - expression level of ITGA7. (**A-F**) shows genes whose expressions were significantly increased in group A (Paralytic esotropia) versus that of group B (Concomitant esotropia). The expression level of MYOD was significantly decreased in group A (Paralytic esotropia) versus that of group B (Concomitant esotropia) (**G**). Significant *P* values are shown as < 0.05 (*), < 0.01 (**), *P* < 0.001(***) and non-significant differences as *p* > 0.05 (#)

To substantiate results from this microarray expression profile and assess differences between the two groups 10 genes (PAX7, MYOD, MYOG, MYF5, CXCR4, PITX1, SIX1, SIX4, VCAM1, ITGA7) related to the differentiation of satellite cells were verified in this report. RNA was extracted from 13 specimens of the paralytic strabismus group and 23 cases of the concomitant strabismus control group. The surgically resected lateral rectus muscle specimens from the two groups were assayed using q RT-PCR for verification and plotted as scatter plots to observe overall expression trends. An independent groups t-test was used to evaluate potential differences in lateral rectus muscle samples from the two groups. Among these, expression levels of PAX7 (2.85 e-5 ± 5.75-e-6) > (3.82E-6 ± 1.68E-7), MYOG (2.16E-4 ± 1.16E-4) > (1.36E-5 ± 1.35E-5), PITX1 (8.98 e-5 ± 1.99E-5) > (2.78e-5 ± 5.56E-6), SIX1 (2.57–3 ± 9.52E-5) > (2.16E-4 ± 1.16E-4), SIX4 (8.57 e-5 ± 3.07E-5) > (4.85E-5 ± 6.60E-6) and ITGA7 (4.54E-4 ± 1.26E-4) > (8.24E-5 ± 6.24E-5) in the paralytic esotropia group were significantly greater than that in the control group (Fig. 3). The expression of MYOD (1.83E-4 ± 3.43E-5) < (6.08E-3 ± 7.54E-4) was significantly lower (*p* < 0.05) in the paralytic esotropia versus control group (Fig. 3).

## Discussion

It is well known that abduction in patients with complete lateral rectus palsy will not recover following routine medial rectus recession and lateral rectus resection. Somewhat surprisingly, we found that maximal medial rectus recession and lateral rectus resection on the affected eye does restore abduction in some of these patients. In this report, we present one such eventuality as shown in a 28-year-old female that experienced complete sixth cranial nerve palsy for 20 years following a traumatic injury. Forced duction test in this case was negative. However, after a 7 mm recession of the medial rectus and 13 mm resection of the lateral rectus in the affected eye, not only was a normal eye position restored, but a nearly normal abduction was also obtained. These findings motivated us to study the possible mechanisms of lateral rectus function recovery in these patients. As surgery on the muscle can contribute to the restoration of abduction in some patients with sixth cranial nerve palsy, we focussed on the muscle and not nerve in this report. A fundamental component for such investigations involves an identification of the molecular background for this effect. Accordingly, we analyzed the gene expression chips in surgically removed lateral rectus specimens of these patients, with special focus on genes related to the activation and inhibition of muscle satellite cells (MSCs).

Muscle differentiation is a complex process which depends on the activation of MSCs [11]. An initial step in this process involves a high expression of the Six1/4 gene, which then promotes expression of the pax7 gene [12]. PAX7, a marker of MSCs [13, 14], mirrors the differentiation potential of MSCs and can also advance the expressions of MYF5 and MyoD, thereby inducing MSCs to develop into myoblasts and triggering initial myoblasts [15]. Myoblasts express myoG, which then promotes cell differentiation processes [9, 10]. Meanwhile, myosin, which is expressed in myocytes, fuses into myotubes to produce contractile functions [16]. Unlike other skeletal muscles, extraocular muscles embody higher concentrations MSCs [17] which, not only possess a strong differentiation ability [9], but also share similar differentiation processes to skeletal muscles.

MyoD can function as a switch for inducing processes involved with MSCs differentiation [18]. In our study, the low expression of MYOD in the paralytic esotropia group suggested that the process of differentiation in the lateral rectus muscle was impaired. Among genes associated with the differentiation process, SIX1 regulates the entire process of differentiation, which includes the expression of PAX7 [19], along with the myogenic regulatory factors (MRF), MYOD and Myogenin [12]. In this report, we observed high expression levels of SIX1/4, PAX7 and MYOG. These findings demonstrate that the most genes critically involved in the process of differentiation (except for the suppression of MYOD) in EOMSCs of patients with paralytic esotropia are active, suggesting that EOMSCs in patients with paralytic esotropia exhibit a relatively effective potential for differentiation.

Although SIX1 regulates the expressions of MYOD, PAX7 and MyoG during differentiation, this gene is, in turn, also regulated by MYOD, PAX7, and MyoG. MYOD and PAX7 may even exert a negative feedback upon SIX1 [20]. Such a mechanism would explain the low expression of MYOD in the lateral rectus muscle of the paralytic esotropia group, while SIX1, PAX7 and MYOG are up-regulated.

PITX1 inhibits muscle regeneration via the p53 pathway and maintains satellite cells in a state of suspension [21]. In this way, an up-regulation of PITX1 has the potential of inhibiting differentiation within the lateral rectus muscle of the paralytic esotropia group. In contrast, Integrin α7 (ITGA7), which facilitates the adsorption of basement membranes in cells, promotes cell proliferation to activate differentiation and regeneration of MSCs [22, 23]. Therefore, an up-regulation of ITGA7 indicates the potential for an effective differentiation process of EOMSCs in patients with paralytic esotropia.

We propose the following series of events as a possible mechanism or the findings that an excessive medial rectus recession plus lateral rectus resection on the affected eyes could restore abduction in some patients. Similar to effects resulting from lateral rectus denervation, the differentiation of MSCs subjected to the local anesthetic, bupivacaine, can be inhibited, thus inactivating the MSCs [24]. However, MSCs in this paralyzed state can be activated and differentiated when damaged by neutrophil infiltration, thereby restoring paralyzed muscle structures to normal [25]. Direct injection of local bupivacaine into EOMs produces significant myonecrosis, which is followed by a relatively rapid regeneration, eventually resulting in myofiber repair and regeneration and a return to normal function [26, 27]. Similarly, we suggest that the loss of function within a completely paralyzed lateral rectus may be due to cessation of the differentiation process and low levels of MYOD expression. However, this completely paralyzed lateral rectus retains the capacity to regenerate and restore partial function as a result of an up regulation in the expressions of SIX1, PAX7 and MYOG and increased differentiation potential induced by stimulation resulting from the surgery. To the best of our knowledge, no research has been directed toward understanding the repair mechanisms of lateral rectus function in patients with paralytic esotropia who underwent surgical correction.

As with any study, there are limitations in this report. First, gene expression within the lateral rectus of patients with paralytic esotropia indicates that MSCs are in an inactive state and that differentiated genes are highly expressed. However, the actual molecular mechanisms and interactions among these factors remain unclear. Second, gene expression levels were not confirmed by tissue staining methods. Third, the actual processes of activation and differentiation of MSCs in vivo after corrective surgery were not determined. Finally, as it was not possible to aquire samples from the nerves of these patients, potential effects upon nerves following surgery that may contribute to these mechaisms remain unknown.

In summary, the gene expression profile of paralytic lateral rectus muscles in patients with complete sixth cranial nerve palsy was investigated. Our findings indicate that under these conditions there is an intiation of processes involved with the regulation of MSCs differentiation, while MSCs remain inactive. At the present time, the reason for this inhibition of MSCs is unknown. The possibility that the functional recovery of the lateral rectus after strabismus surgery is due to the activation of MSCs by surgical stimulation warrants further study in animal models.

## Supplementary Information

**Additional file 1.**

**Additional file 2.**

## Data Availability

All data generated or analysed during this study are included in this article and its supplementary information files.
